# Supplementary material for: Silk-enabled conformal intraventricular interfaces for minimally invasive neural recordings
Source: Nat Commun. 2025 Oct 23;16:9366. doi: 10.1038/s41467-025-64397-9 (PMC12549829; doi:10.1038/s41467-025-64397-9)
Supplement: Supplementary file 1 — Supplementary Information [file 41467_2025_64397_MOESM1_ESM.pdf]

Supplementary Information for

Silk-enabled Conformal Intraventricular Interfaces for Minimally Invasive Neural Recordings

Jizhi Liang, Xiner Wang, Zhaohan Chen, Xiaoling Wei, Liuyang Sun, Keyin Liu, Zhifeng Shi, Tiger H. Tao* and Zhitao Zhou*

Corresponding author: Tiger H. Tao, tiger@mail.sim.ac.cn; Zhitao Zhou, ztzhou@mail.sim.ac.cn

The PDF file includes:

Supplementary Note 1 to 3
Supplementary Fig. 1 to 25

Supplementary Note 1 | IVI self-unfolding mechanism

According to the established theory of shape memory polymers[1], the β-sheet structures in the silk fibroin scaffolds act as net points, while the weak forces, such as hydrogen bonds serve as reversible switches. Initially, the silk scaffold is in a dry, isotropic, and glassy state. Upon the pre-hydration process, these switches open as the silk scaffold swelling, providing the IVI with the capability for elastic deformation, which leads to the temporary orientation and immobilization of α-helix and random coil in the direction of external force during the miniaturization process, thus forming the top compression part and the bottom tension part. When immersed in liquid, these switches open again, and oriented secondary structures of the top part release and expand, while those of the bottom part release and contract, reverting to their original elastomeric states toward the opposite direction.

Supplementary Note 2 | Electrostatic shielding for electrophysiology

The environment within deep brain regions is vastly more intricate than that of cortical surface or intracortical layers, where noise attenuation during surgery is more manageable via a variety of grounding and proximal referencing techniques. Moreover, due to the requirement for use within the operating room environment and in conjunction with minimally invasive implantation tools, the need for electrostatic shielding at the neural interface end to ensure signal fidelity is pronounced. The complexity of electrical noise in such settings necessitates cumbersome measures, including isolated grounding, Faraday cages, and the suspension of certain medical equipment[2], which are impractical for IVI application during surgery. Herein, we advocate for integrating in-plane electrostatic shielding within the IVI, which not only simplifies its intraoperative use but also enhances signal stability and clinical applicability. Traditional shielding approaches, however, often entail bulky extra-layer designs that hinder the device's ability to conform to neural tissues, particularly within the deep brain regions.

Supplementary Note 3 | An effectiveness study in diagnosis of Parkinson's disease on sheep models by IVI

Due to the structural similarity of the sheep brain to the human brain, including comparable subcortical anatomical structures, they serve as ideal candidates for translational research of basal ganglia disorders (including PD)[3]. A widely studied electrophysiological biomarker of PD is the β-burst activity found within the pathological neural networks of the basal ganglia, particularly in brain regions like the subthalamic nucleus[4]. Notably, the striatum serves as the primary input structure of the basal ganglia, with the dorsolateral striatum (including the caudate nucleus and putamen) forming part of a semi-closed neural circuit with the cortex. We hypothesize that the caudate nucleus is also involved in the pathological neural activity of the basal ganglia in PD and may provide meaningful information for decoding and state discrimination of the pathological neural networks in PD. There are primarily two types of experimentally induced Parkinsonian models in sheep. The first is an acute intraoperative model that mimics Parkinsonian symptoms through the acute intracerebral injection of cholinergic drugs. The second type is a chronic model induced by long-term drug infusion[5], such as the chronic Parkinsonian sheep model resulting from nigral dopaminergic depletion induced by MPTP (1-Methyl-4-phenyl-1,2,3,6-tetrahydropyridine). We initially embarked on applying IVI in an acute Parkinsonian sheep model to validate its feasibility. Previous investigations have indicated that only intracerebral injections of substantial

doses of carbachol are capable of inducing Parkinsonian symptoms[6]. After initiating recordings from the striatum using the IVI, we sequentially administered a small dose of 5 μg carbachol followed by a large dose of 20 μg carbachol to the dorsolateral striatum. Statistical analysis of the changes in striatal beta energy confirmed that only a large dose of intracerebral carbachol injection could induce an increase in striatal beta energy (**fig. S13 to S15**).

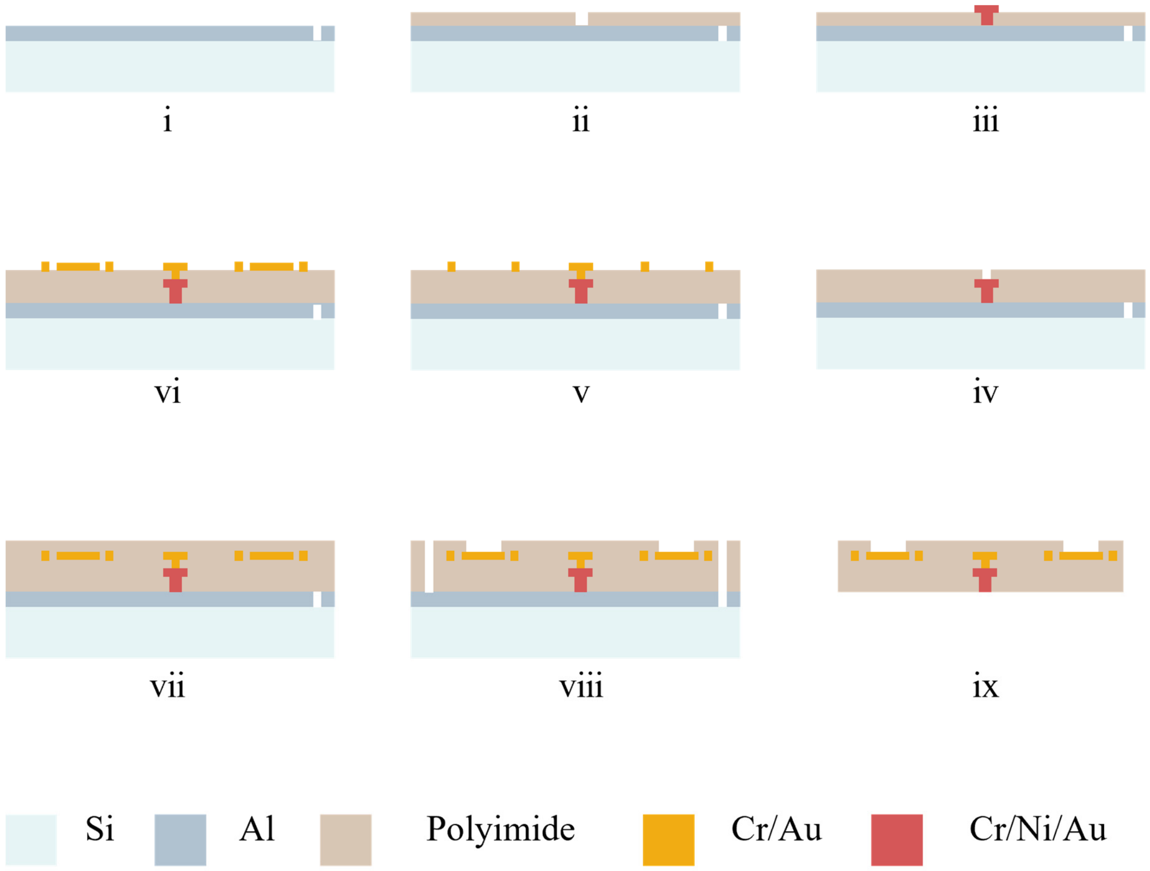

Supplementary Fig. 1

Fabrication process flow chart of Intraventricular Interfaces (IVI) microelectrodes. A few points need to be noted in particular. We incorporated multilayer interconnection technology to establish dependable electrical connections between the reflow soldering pads and electrode sites on the opposing surface. After the photolithography patterning and wet etching of the PI substrate (ii), the reflow soldering pads metal layer (Cr/Ni/Au, 100/1000/5000Å) was deposited using electron beam evaporation (iii). The in-plane metal shielding layer (Cr/Au, 150/4000Å) is the same as multilayer interconnecting vias and interconnectors, and was fabricated simultaneously (v).

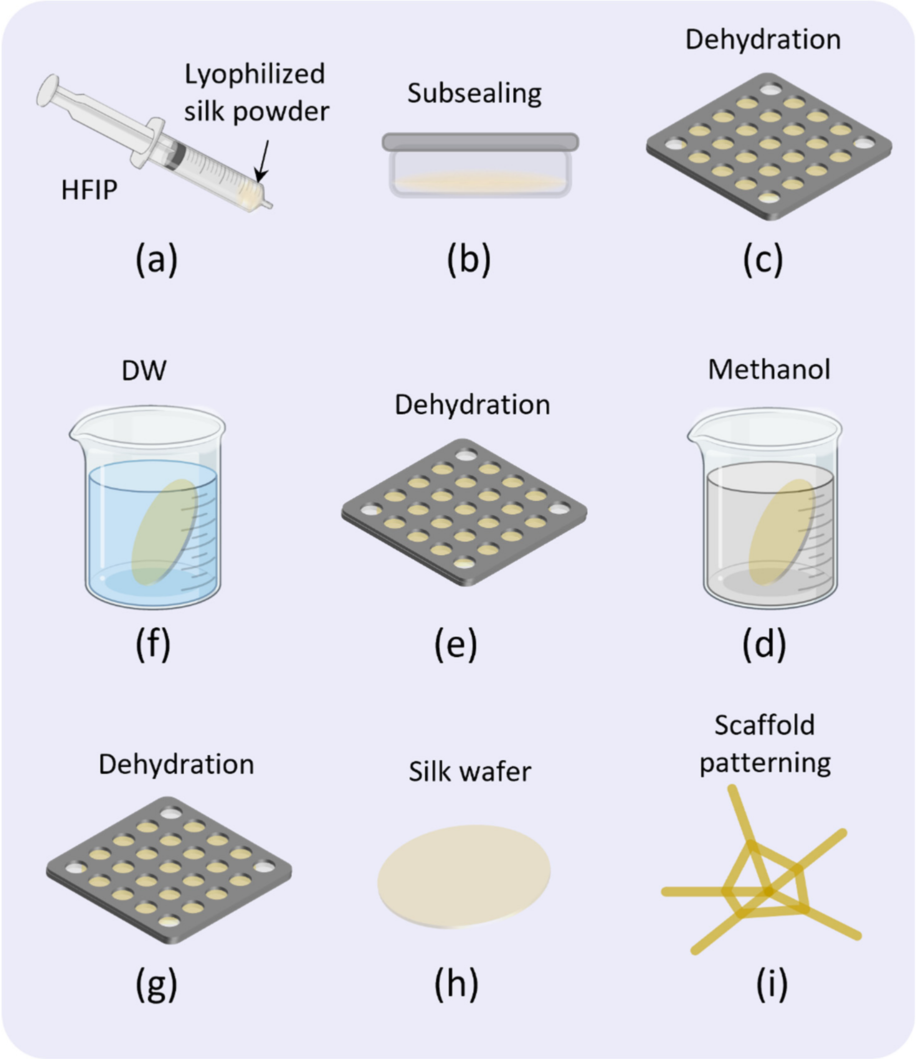

Supplementary Fig. 2

Fabrication process flow chart of silk scaffold. Lyophilized silk fibroin powder was dissolved in HFIP solution, with the volume determined by the desired film thickness and density (1.3 g/cm^3). This solution was then cast in a polyethylene container under sub-seal conditions for smooth evaporation. After drying, the film underwent methanol and water treatments to remove solvents, followed by flattening. Laser processing patterned the silk wafer, preparing it for assembly use after immersion in water.

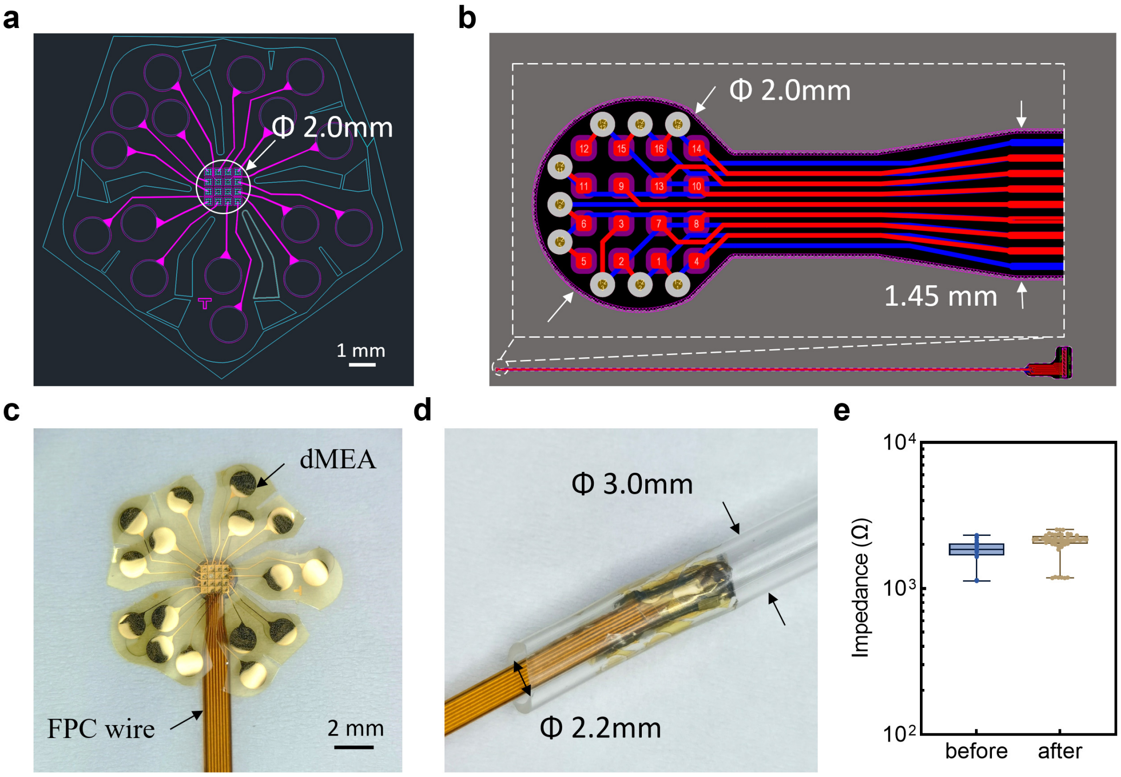

Supplementary Fig. 3

Small-size intraventricular Interfaces (IVI). a The layout design of the small size deformable microelectrode array. **b** The corresponding small size flexible printed circuit board. **c** The image of small size IVI device. **d** The image of small size IVI integrated into medical catheter with inner diameter of 2.2 mm. **e** Impedance tests of IVI before and after the catheter assembly (n = 48 electrode sites from 3 devices).

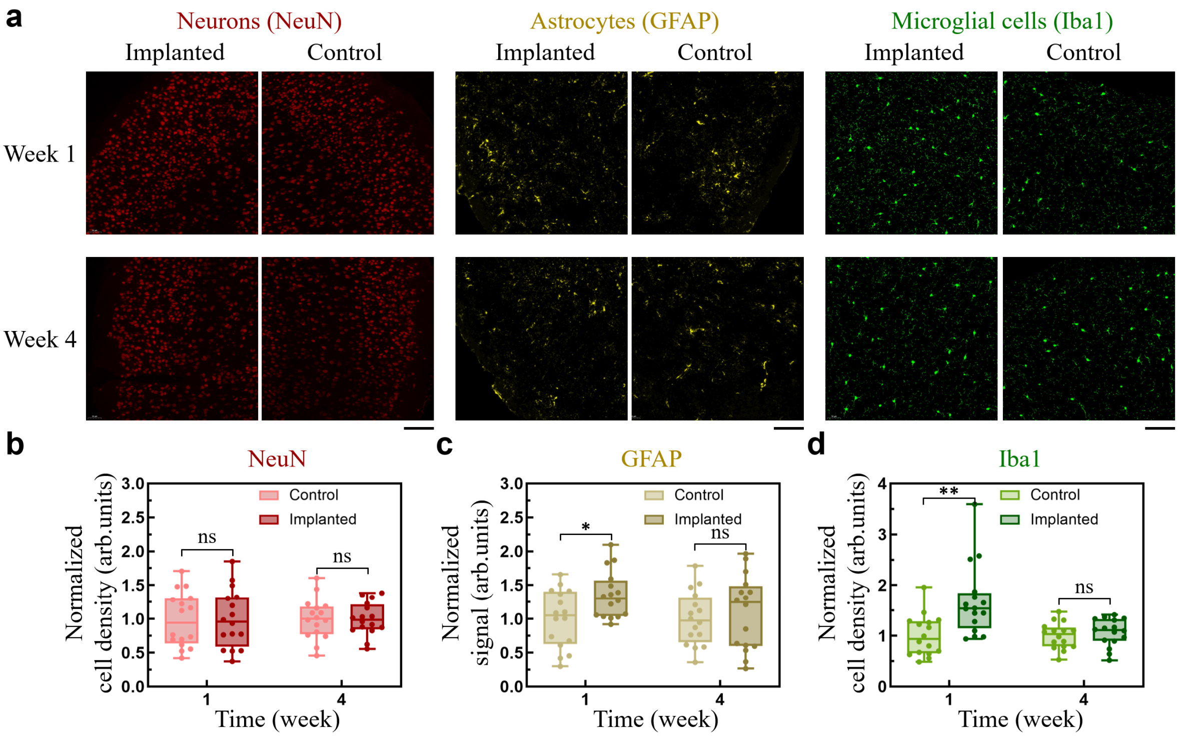

Supplementary Fig. 4

Biocompatibility of IVI for neural tissues. a Immunohistochemistry studies on mice with the IVI implanted epidurally. Example immunofluorescence images of a mice cortex under the IVI (implanted) and on the contralateral control side (control) at different time points after implantation. Scale bar, 100 μm. **b** Normalized cell density of NeuN-labelled neurons. **c** Normalized signal of GFAP-labelled astrocytes. **d** Normalized cell density of Iba1-labelled microglial cells. The box plots show the median and quartile range. $n = 16$ from 3 mouse for week 1 and 4 data. For the data that could be represented by a normal distribution, two-sided unpaired t tests were used. ns, $p > 0.033$, $*p < 0.033$, $**p < 0.002$ and $***p < 0.001$. $p = 0.0168$ (week 1, **c**), $p = 0.0022$ (week 1, **d**). The box plots show the median and interquartile range, and the whiskers denote 1.5× the interquartile range. Source data are provided as a Source Data file.

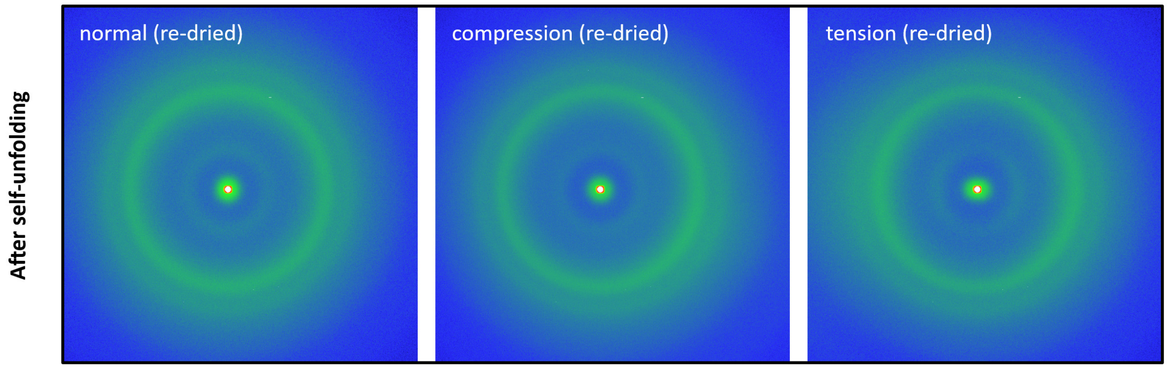

Supplementary Fig. 5

Optical characterization of silk-scaffolds after self-unfolding. The 2D-WAXD patterns of the re-dried samples showed that the diffraction arcs in the compression and tension parts caused by oriented crystallization revert to diffraction rings after self-unfolding, closely resembling the diffraction ring patterns of the normal group samples.

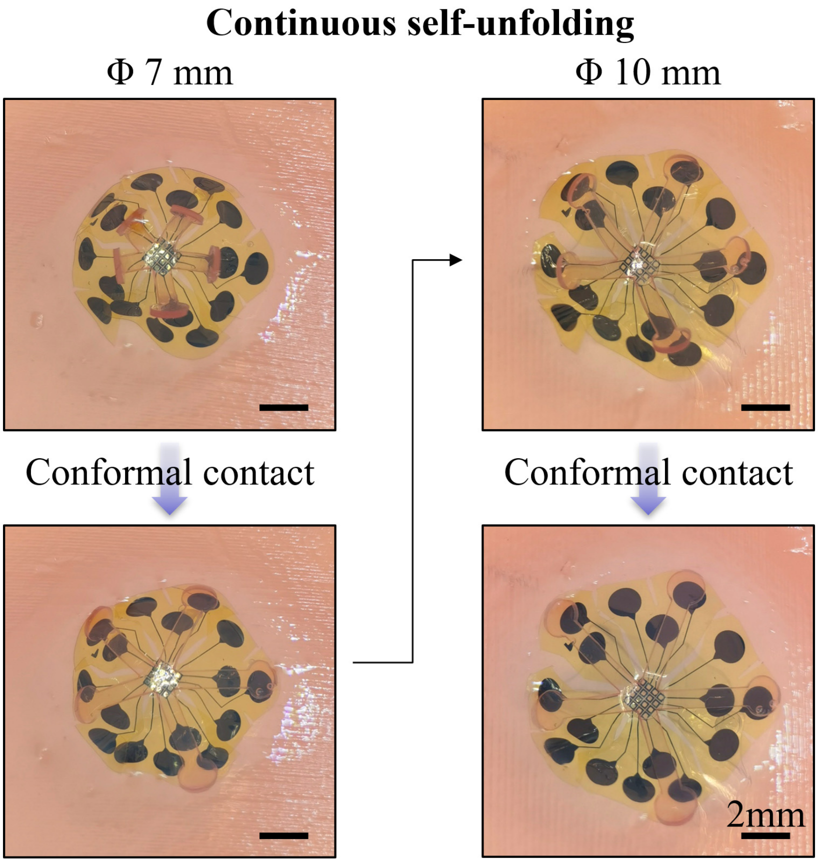

Supplementary Fig. 6

In vitro self-unfolding and conformal attachment validations of IVI on agarose brain fantom model with different curvatures.

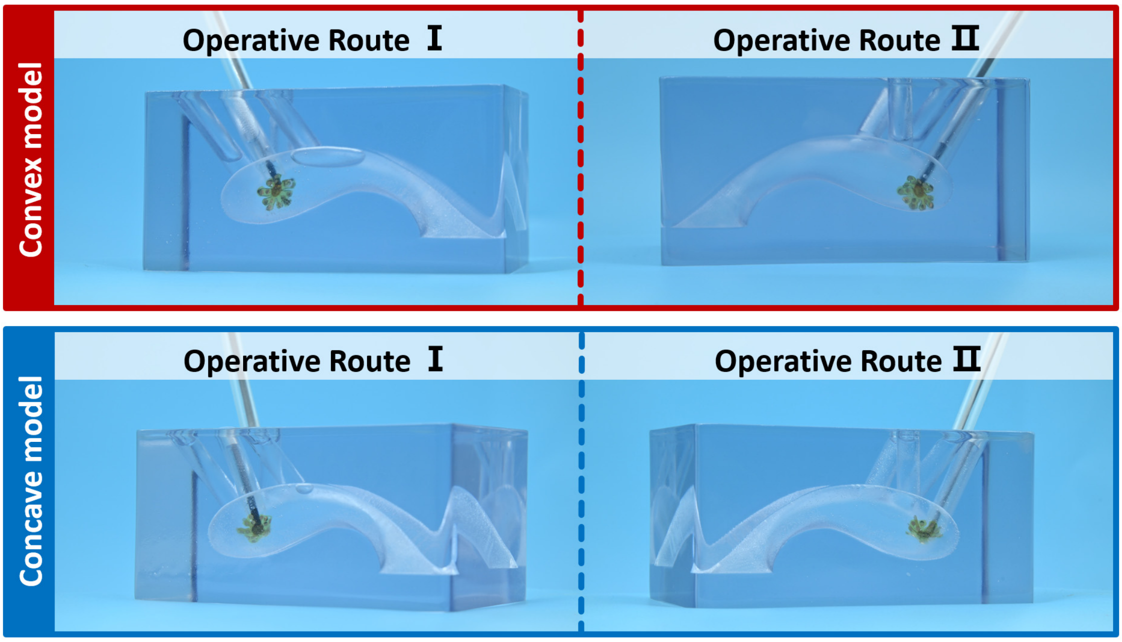

Supplementary Fig. 7

Implantation validation in transparent lateral ventricle *in vitro* model. Top: Implantation validation of the convex model of IVI implanted in transparent lateral ventricle *in vitro* model with different implantation routes. Bottom: Implantation validation of the concave model of IVI implanted in transparent lateral ventricle *in vitro* model with different implantation routes. The model was infused with CSF heated to 37°C to mimic the real cerebroventricular environment.

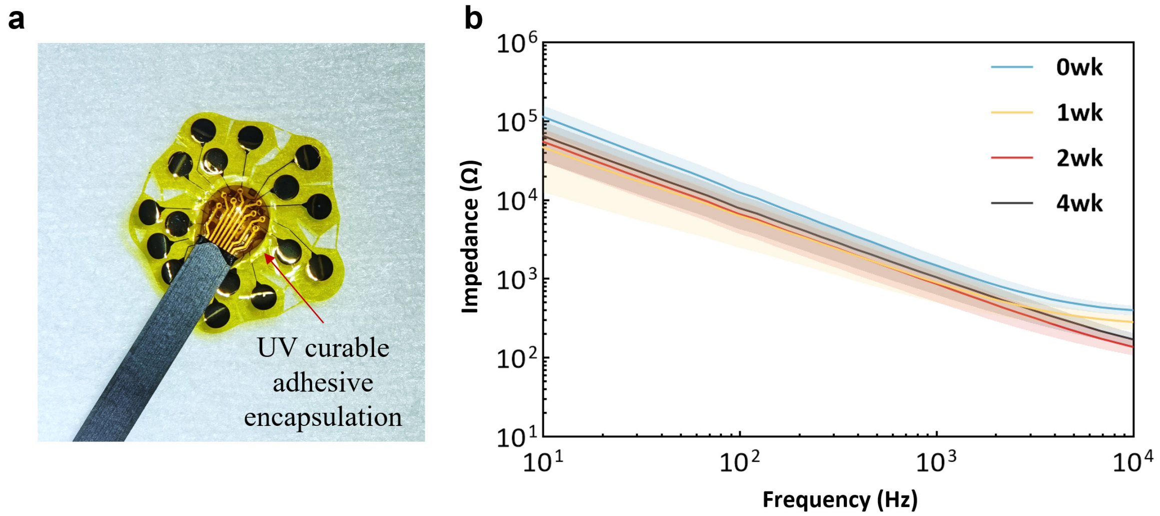

Supplementary Fig. 8

Packaging of the dMEA. a The image of the ultra-violet curable adhesive encapsulation of the dMEA and the FPC. **b** Stable encapsulation in artificial CSF at 37°C enduring 4 weeks verified by impedance spectrum tests at 1st week, 2nd week and 4th week after the immersion (*n* = 24 electrode sites). Data are presented as mean values ± SD. Source data are provided as a Source Data file.

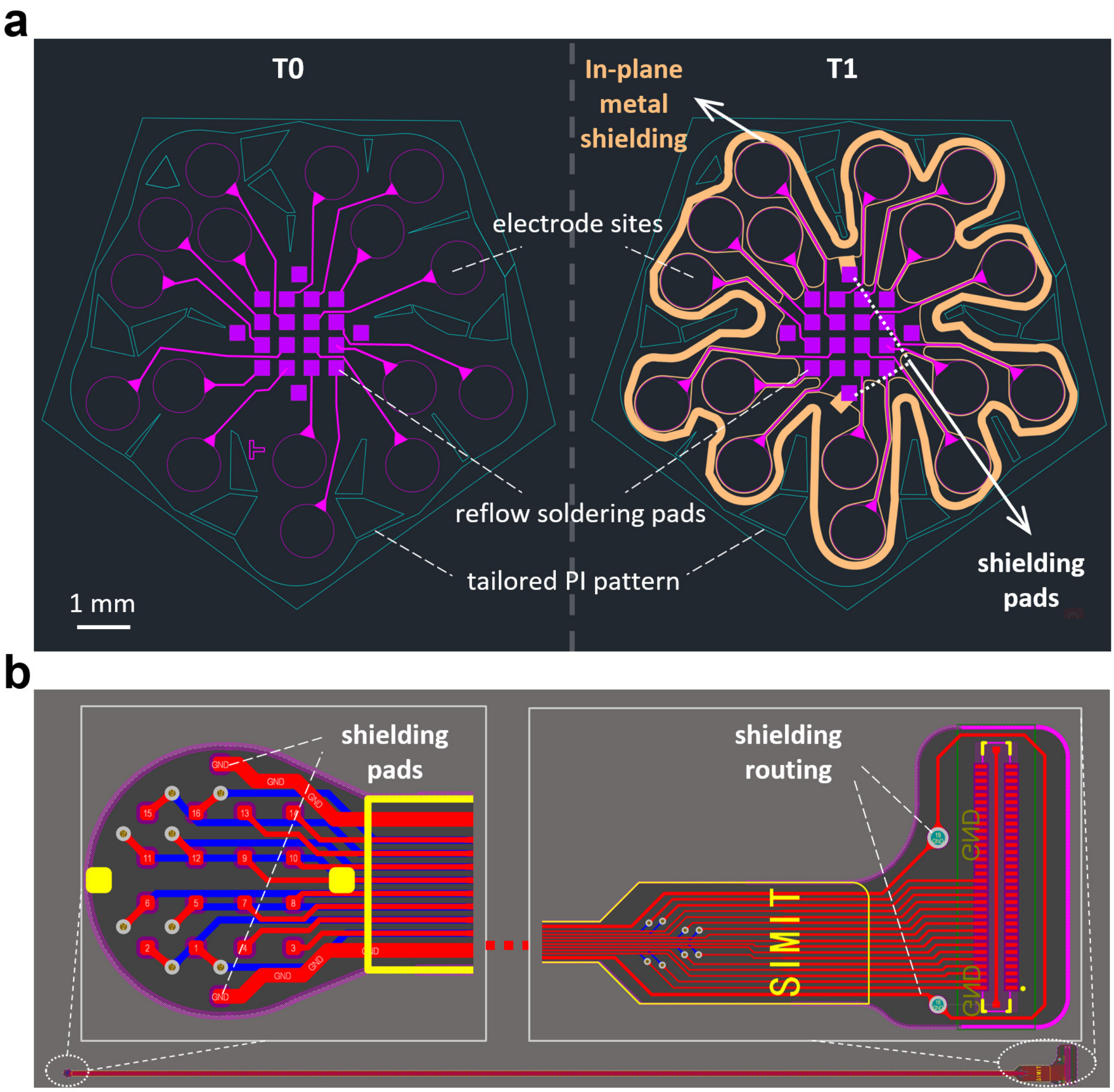

Supplementary Fig. 9

Deformable microelectrode array (dMEA) and in-plane shielding design. a The layout design of T0- and T1-types of IVI. The in-plane metal shielding layer comprised closed inner bell-like and outer irregular ring-like shielding traces which were shorted to the shielding pads connected to the shielding routing and the headstage ground. **b** The metal shielding layer of the dMEA needs to be connected to the ground (GND) of the headstage through shielding pads and shielding routing to achieve the proper electrostatic shielding effect.

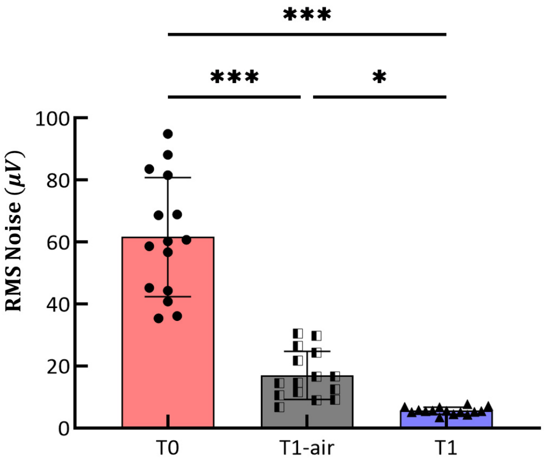

Supplementary Fig. 10

In vitro validation of the in-plane metal shielding. Bar plots showed the root mean square (RMS) values of the raw data recorded by T0, T1 and T1-air (denotes the configuration with the shielding routing leads hanging freely). T1 refers to the effective grounded configuration of IVI. Ordinary one-way analysis of variance (ANOVA), $p = 0.0000$, T0 versus T1-air, $p = 0.0000$; T0 versus T1, $p = 0.0000$; T1 versus T1-air, $p = 0.0389$. Source data are provided as a Source Data file.

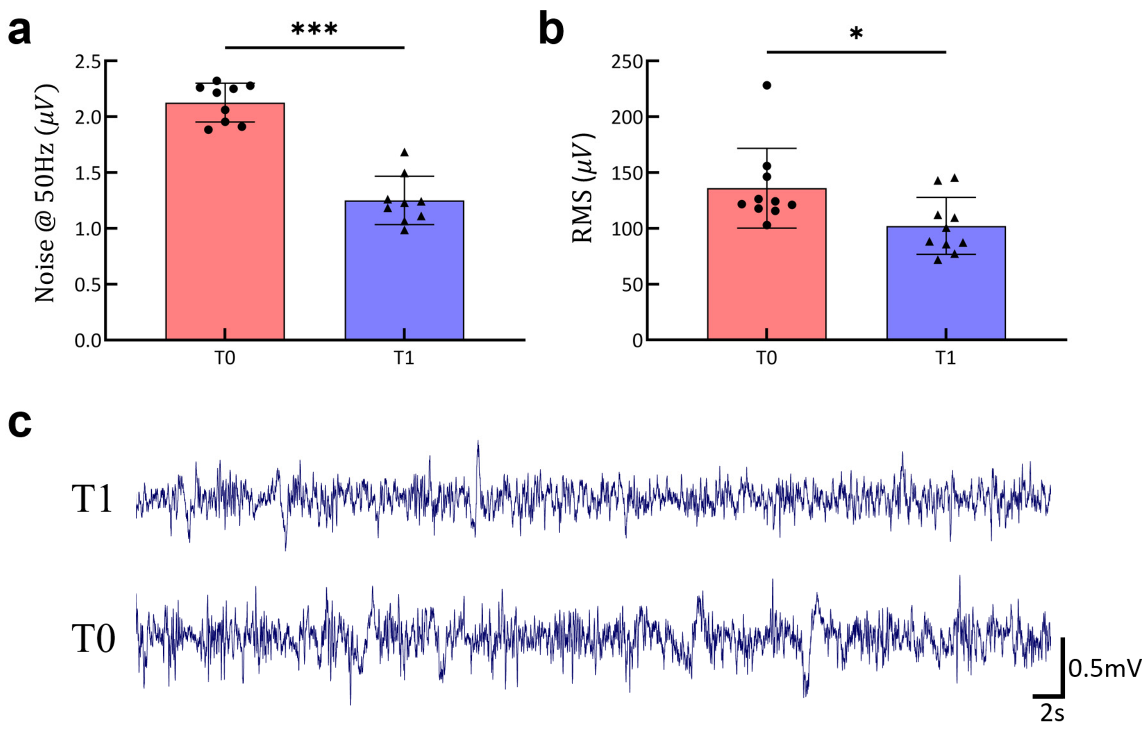

Supplementary Fig. 11

Acute recording of subdural electrophysiological signals in an anesthetized Labrador dog. a Bar plots showed the power line noise at 50 Hz of T0- and T1-types of IVI. Unpaired two-tailed t test: $p = 0.0000$. **b** Bar plots illustrated a statistical analysis comparing the RMS values of the raw data recorded by T0 and T1. Unpaired two-tailed t test: $p = 0.0267$. **c** Typical time-domain waveforms of the raw neural signals recorded by T0 and T1. Source data are provided as a Source Data file.

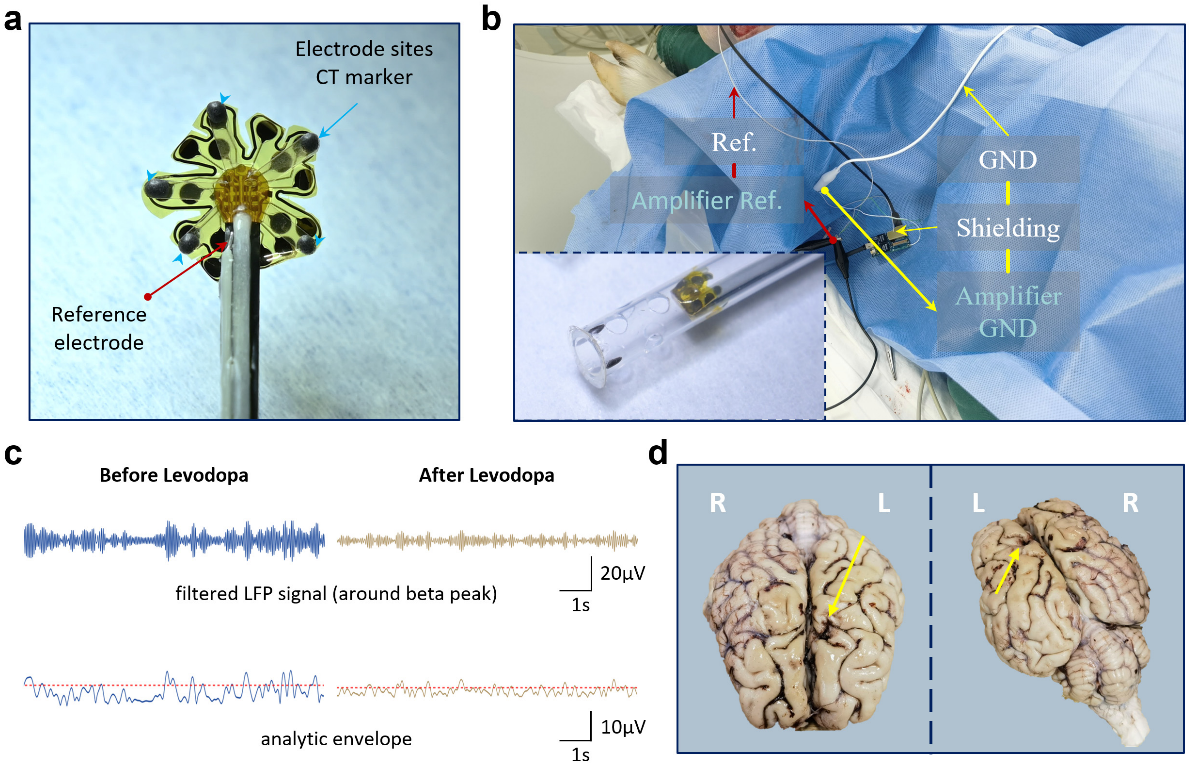

Supplementary Fig. 12

Application of IVI in the parkinsonian sheep. a A photo of the IVI used in the parkinsonian sheep model. **b** A photo of final setup of the ground and reference configurations of the IVI in the CT operating room. Particularly, the shielding routing of the IVI was shorted with the skull screw and the ground on the headstage. The inset photo shows the IVI standing ready for use in the operation. **c** Typical beta band neural activity and analytic amplitude (envelope) recorded by IVI on the caudate nucleus surface of parkinsonian sheep before and after intraoperative levodopa medication. **d** Photos of the brain dissected from the euthanized sheep. Yellow arrows show the puncture site of minimally invasive implantation of IVI.

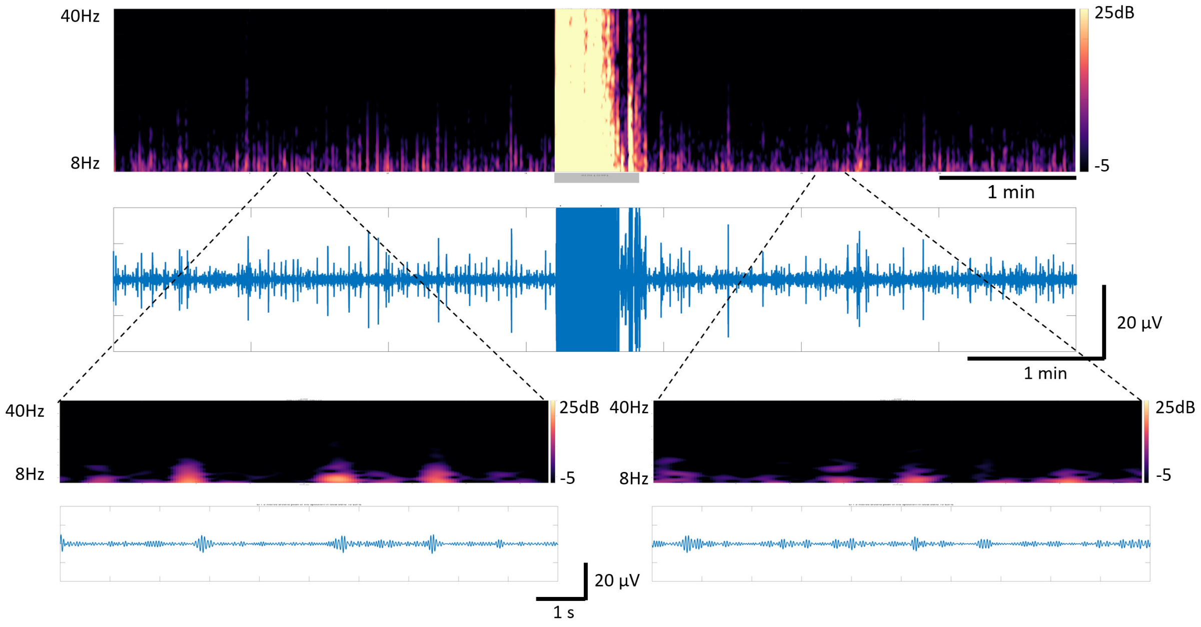

Supplementary Fig. 13

The spectrograms and waveforms of caudate surface neural activity recorded by IVI before and after the injection of 5 μg carbachol in the acute Parkinsonian sheep. Time-frequency analysis only indicated mechanical artifacts introduced during 5 μg carbachol intracerebral injection and no notable power change in beta band.

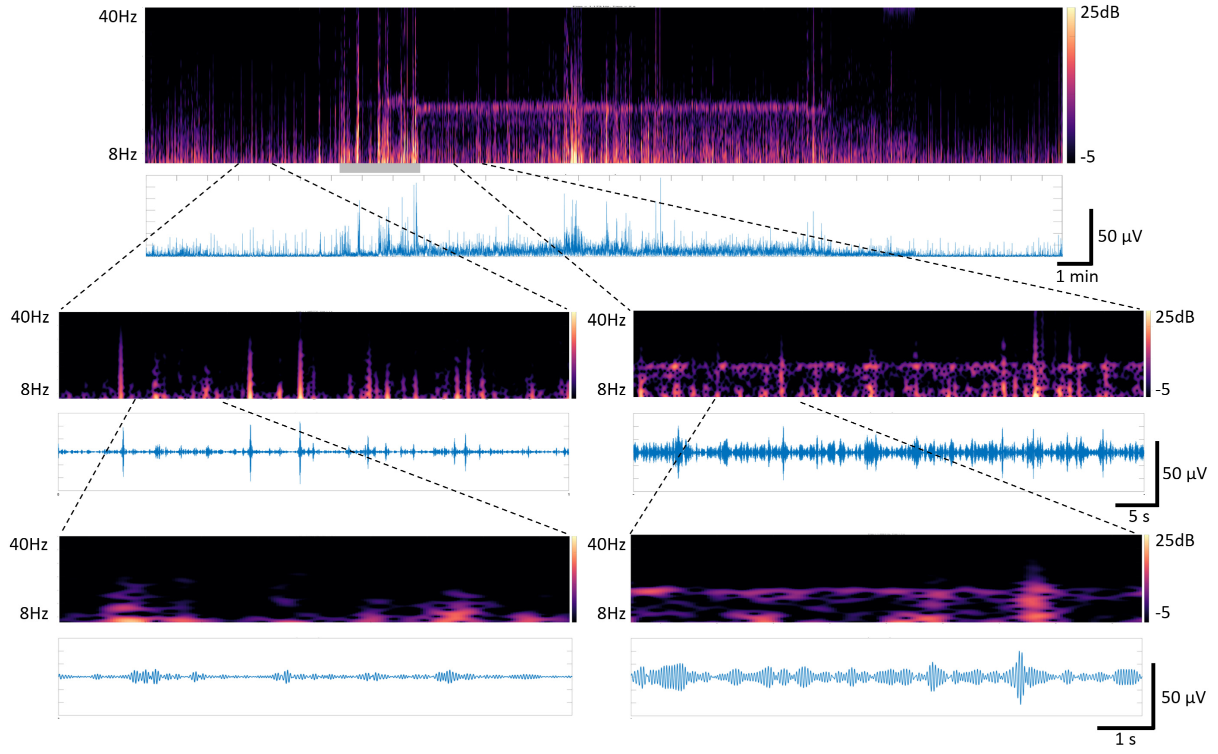

Supplementary Fig. 14

The spectrograms and waveforms of caudate surface neural activity recorded by IVI before and after the injection of 20 µg carbachol in the acute Parkinsonian sheep. Time-frequency analysis showed an immediate increase in beta power lasting several minutes post-injection.

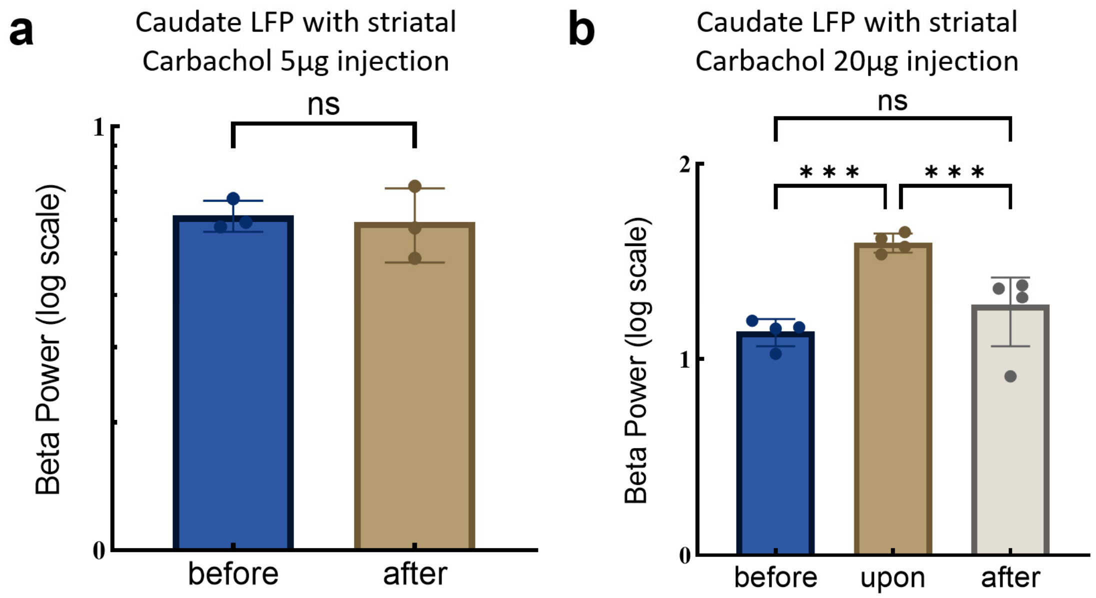

Supplementary Fig. 15

Beta power changes in acute parkinsonian sheep. a Scatter dot plot showed no significant change occurred in beta power of caudate during the 5 µg Carbachol striatal intracerebral injection (*n* = 3 recordings). Unpaired two-tailed t-test: $p = 0.7967$. **b** Scatter dot plot showed a significant change occurred in beta power of caudate upon the 20 µg Carbachol striatal intracerebral injection, and the beta power returned to the normal state eventually. Ordinary ANOVA (*n* = 4 sessions of recordings), $p = 0.0001$, before versus upon, $p = 0.0001$; after versus upon, $p = 0.0007$; before versus after, $p = 0.3684$. ns, nonsignificant. Source data are provided as a Source Data file.

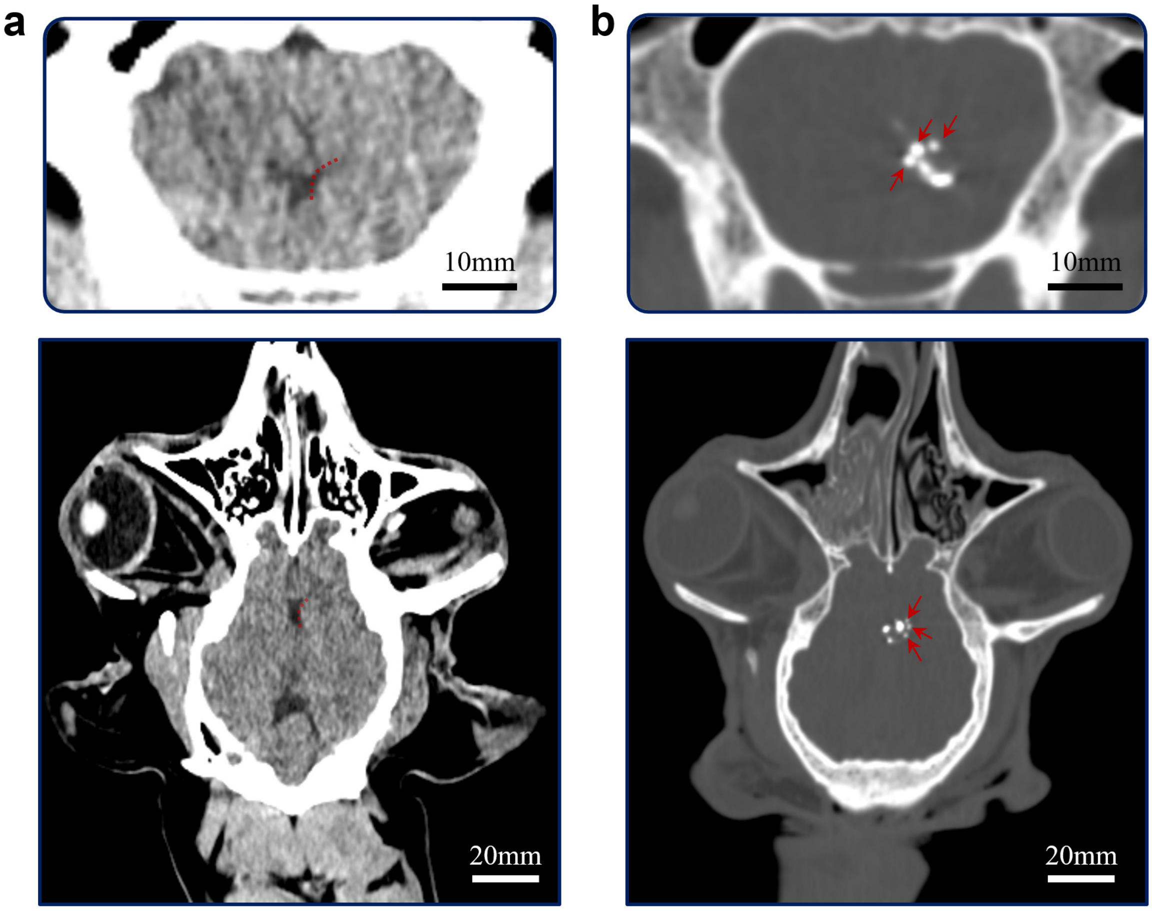

Supplementary Fig. 16

CT scans of the chronic Parkinsonian sheep. a Preoperative CT scans of the chronic Parkinsonian sheep model in the coronal and axial planes. Red dashed lines indicate the contour of the head of the caudate nucleus. **b** Intraoperative CT scans of the chronic Parkinsonian sheep model in the coronal and axial planes. Red arrows indicate the CT markers on the IVI.

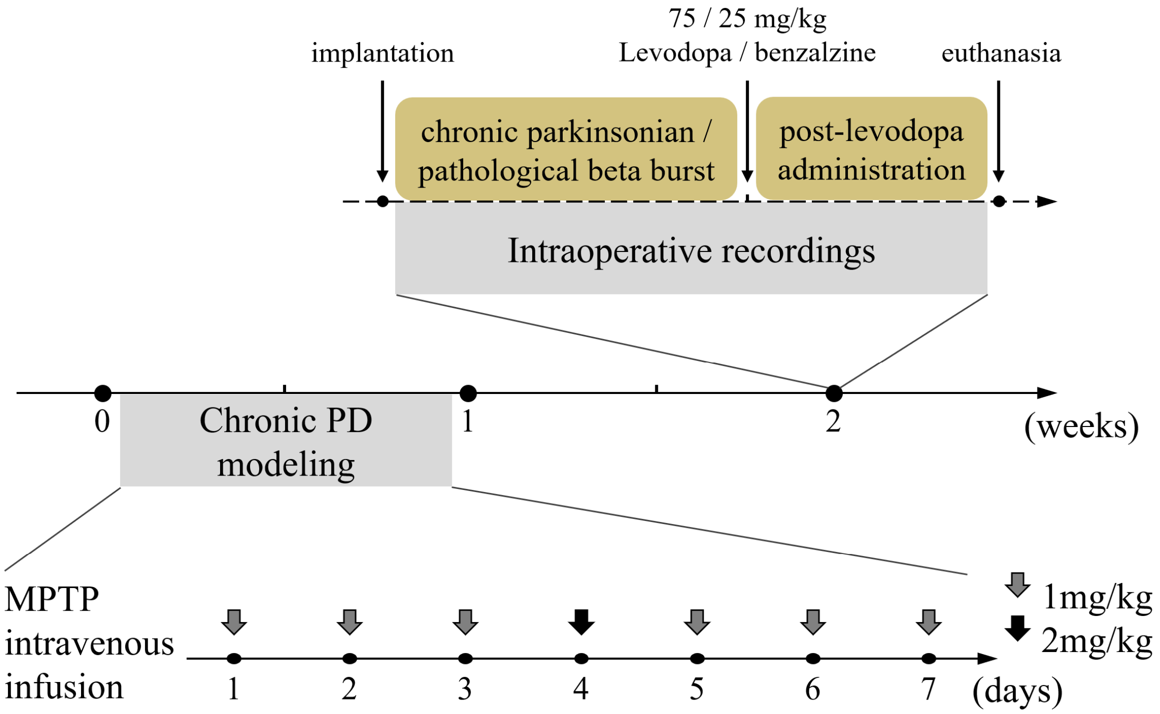

Supplementary Fig. 17

Experiment scheme of the intraoperative recordings in parkinsonian sheep model.

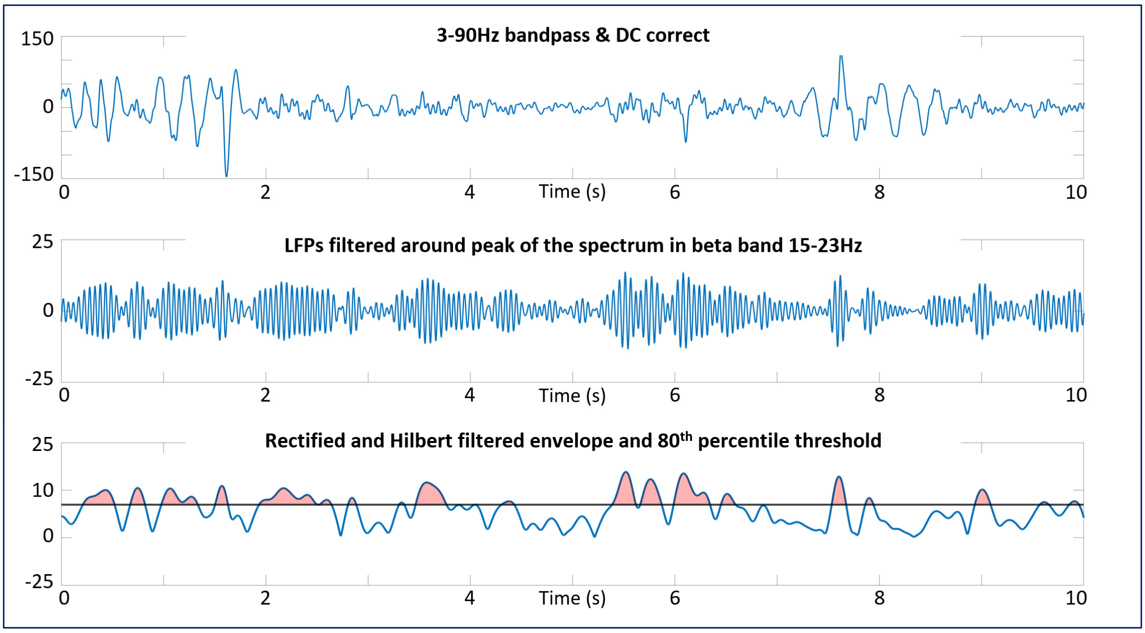

Supplementary Fig. 18

Beta burst calculation method. Bursts were detected by applying a threshold (80th percentile) to the signal envelope obtained by rectification and Hilbert filtering of the caudate head surface local field potentials around the peak of the spectrum in the beta band.

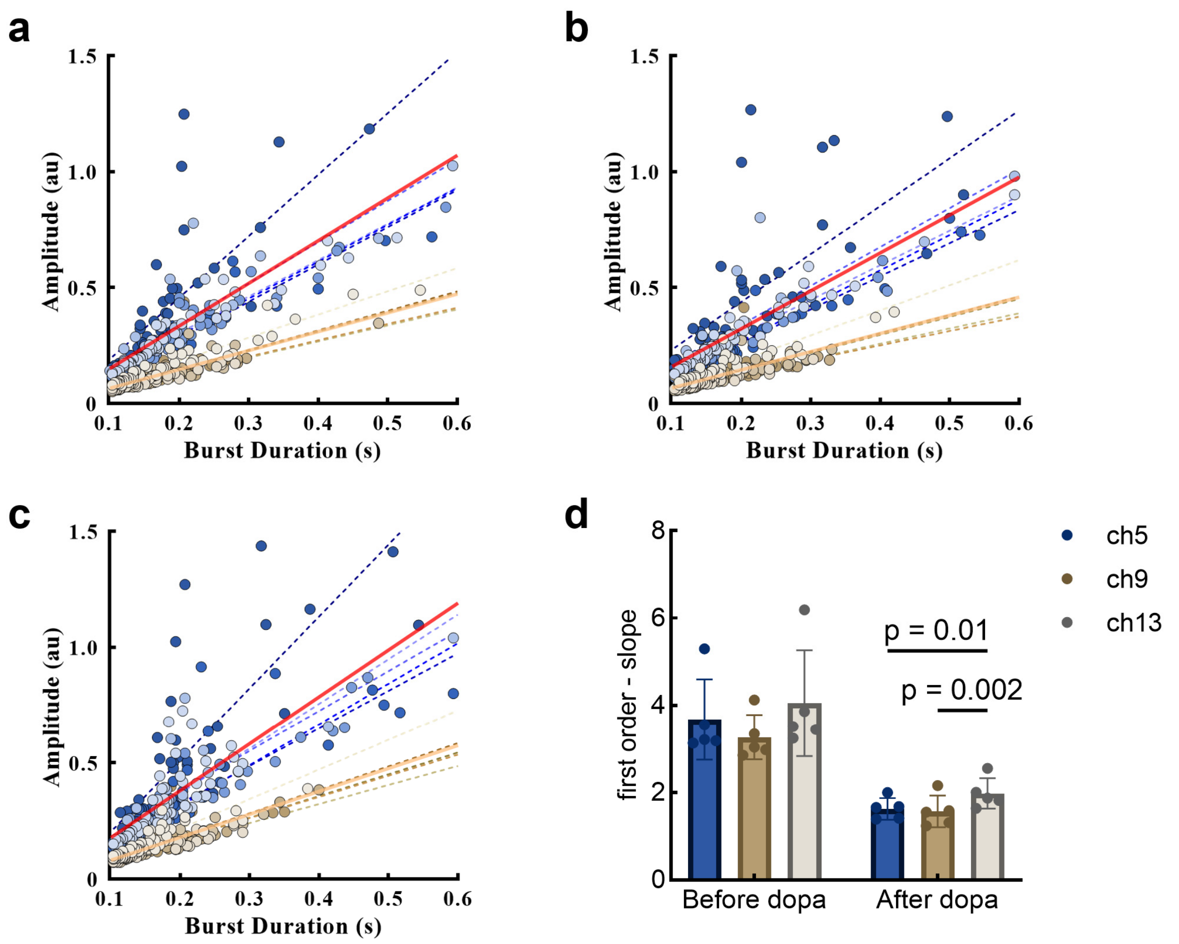

Supplementary Fig. 19

Statistical analysis of multichannel beta burst amplitude and duration. a-c Scatter diagrams of beta bursts durations and amplitude of channel 5, channel 9 and channel 13. Each diagram comprises five minutes effective recordings, and first order fitting analysis was conducted on every minute recordings as represented by a dotted line, and the solid line represent the average result of first order fitting analysis. Scatters in blue refers to the before levodopa condition (Before LD) and scatters in brown refers to the after levodopa condition (After LD). **d** Grouped scatter dot plots and statistical analysis on the first order slope of the two conditions across different IVI channels. n = 5 sessions of recordings. p = 0.0101 (ch5-ch13), p = 0.0022 (ch9-ch13). Two-way ANOVA test were used. Source data are provided as a Source Data file.

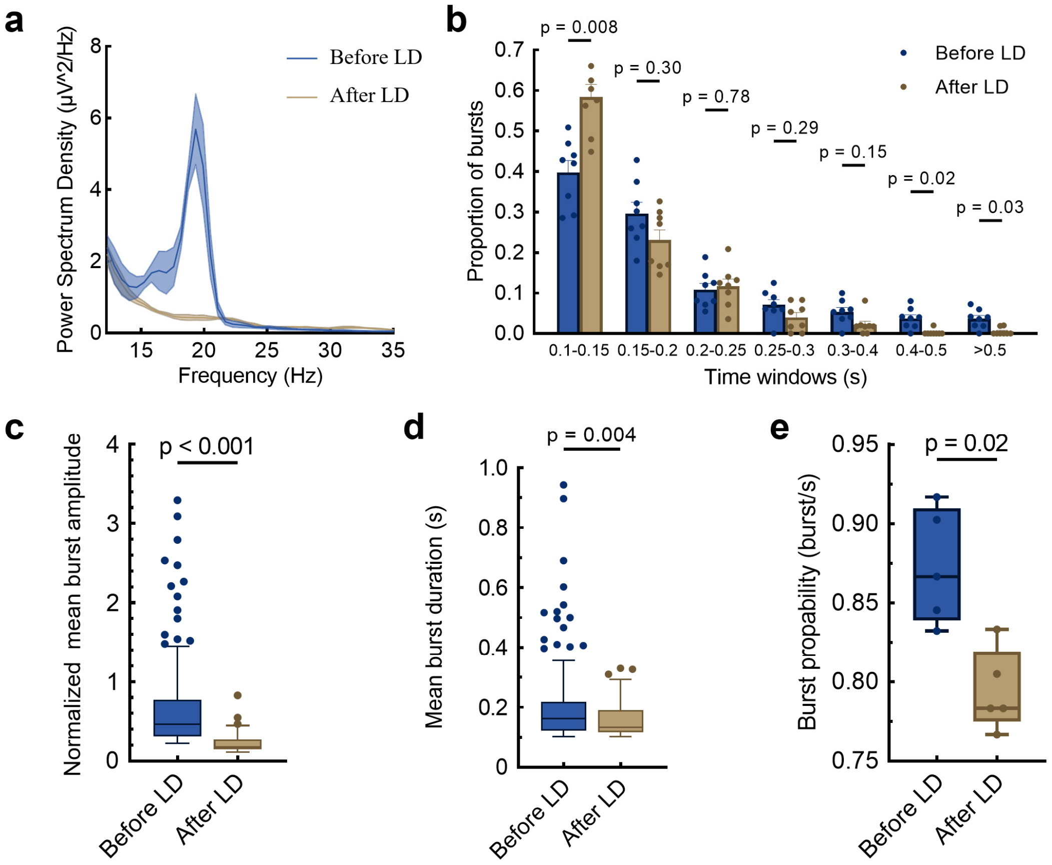

Supplementary Fig. 20

Beta bursts statistical analysis for channel 9. a Power density spectral of local field potentials recordings (n = 4 sessions of recordings). **b** Bar plots showing changes in distribution of burst durations as a percentage of total number of bursts during Before LD and After LD conditions. n = 8 sessions of recordings. Multiple Mann-Whitney tests were used. p = 0.0076, 0.2953, 0.7803, 0.2932, 0.1504, 0.0231 and 0.0330, from left to right group. **c** The mean burst amplitude during Before LD and After LD. p = 0.0000. **d** The mean duration of all bursts during Before LD and After LD. p = 0.0037. **e** The probability of bursts to occur (illustrated as burst/s) during Before LD and After LD. p = 0.0159. Mann-Whitney test were used for (**c-e**). Data are presented as mean values + SEM in (**b**). The box plots show the median and interquartile range, and the whiskers denote 1.5× the interquartile range. Source data are provided as a Source Data file.

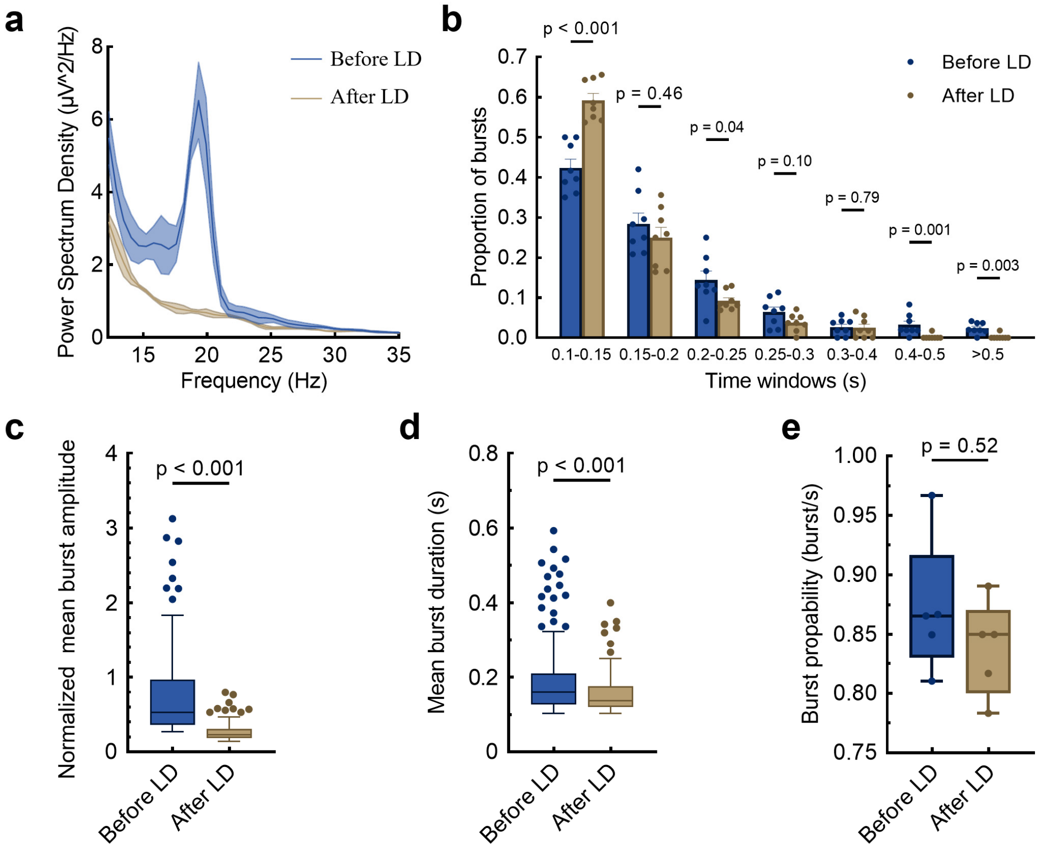

Supplementary Fig. 21

Beta bursts statistical analysis for channel 13. a Power density spectral of local field potentials recordings (n = 4 sessions of recordings). **b** Bar plots showing changes in distribution of burst durations as a percentage of total number of bursts during Before LD and After LD conditions. n = 8 sessions of recordings. Multiple Mann-Whitney tests were used. p = 0.0002, 0.4580, 0.0406, 0.0985, 0.7941, 0.0014 and 0.0025, from left to right group. **c** The mean burst amplitude during Before LD and After LD. p = 0.0000. **d** The mean duration of all bursts during Before LD and After LD. p = 0.0005. **e** The probability of bursts to occur (illustrated as burst/s) during Before LD and After LD. p = 0.5238. Mann-Whitney test were used for (**c-e**). Data are presented as mean values + SEM in (**b**). The box plots show the median and interquartile range, and the whiskers denote 1.5× the interquartile range. Source data are provided as a Source Data file.

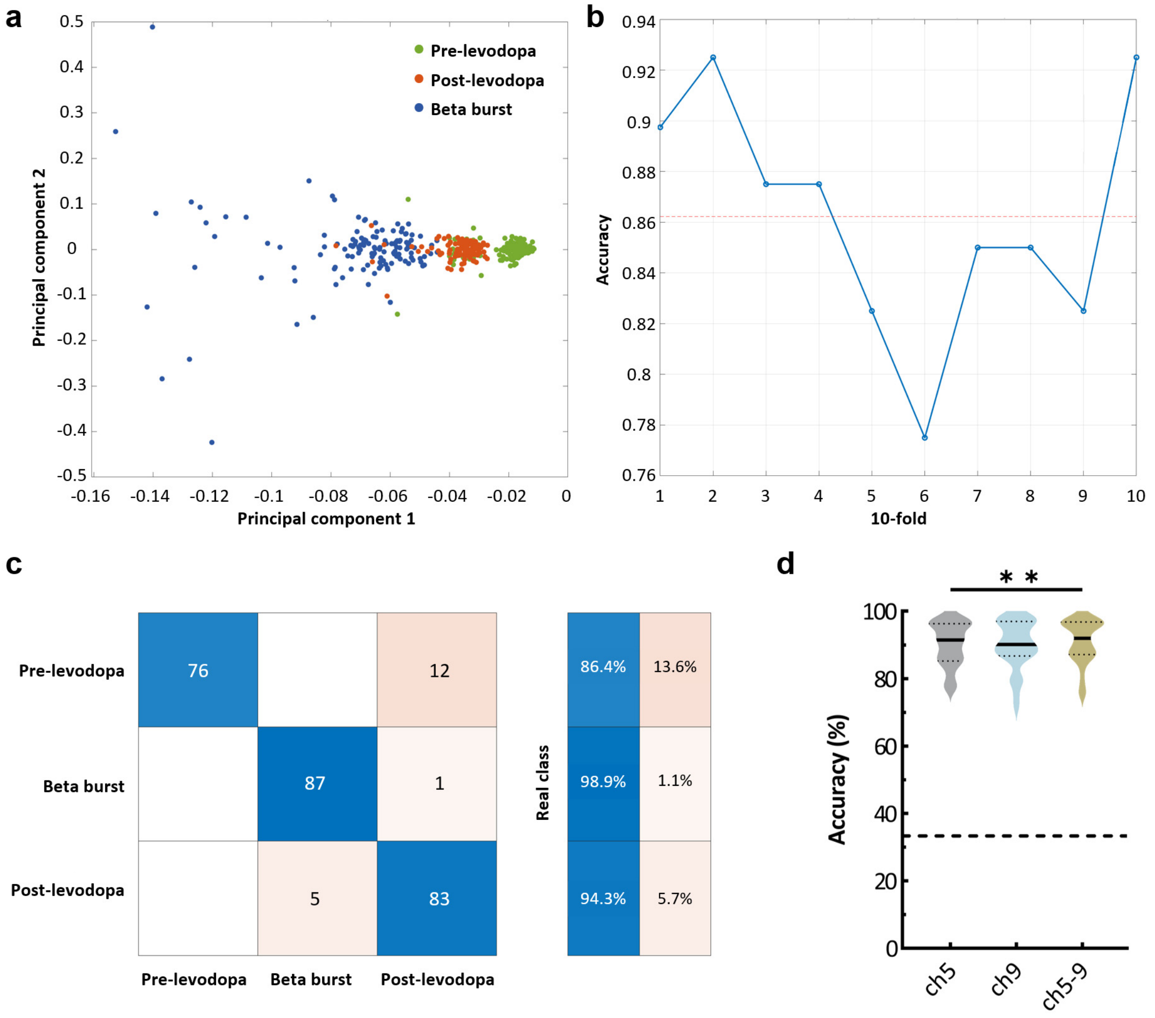

Supplementary Fig. 22

Neural activity discrimination model and high-accuracy neural activity discrimination. a Two-dimensional feature space obtained by singular value decomposition (SVD) under one typical random data set partitioning. **b** Ten-fold cross-validation of linear discriminant analysis (LDA) training under one typical random data set partitioning. **c** Confusion matrix showed validation result of SVD-LDA model on the test set under one typical random data set partitioning, and the accuracy was 93.18%. **d** Violin plot showed the accuracy increase to 91% when combining data sets of channel 5 and channel 9 for neural activity discrimination. Wilcoxon test were used. p = 0.0070. n = 30 random partitions and tests. The truncated violin plots show the median and interquartile range, and the whiskers denote 1.5× the interquartile range. Source data are provided as a Source Data file.

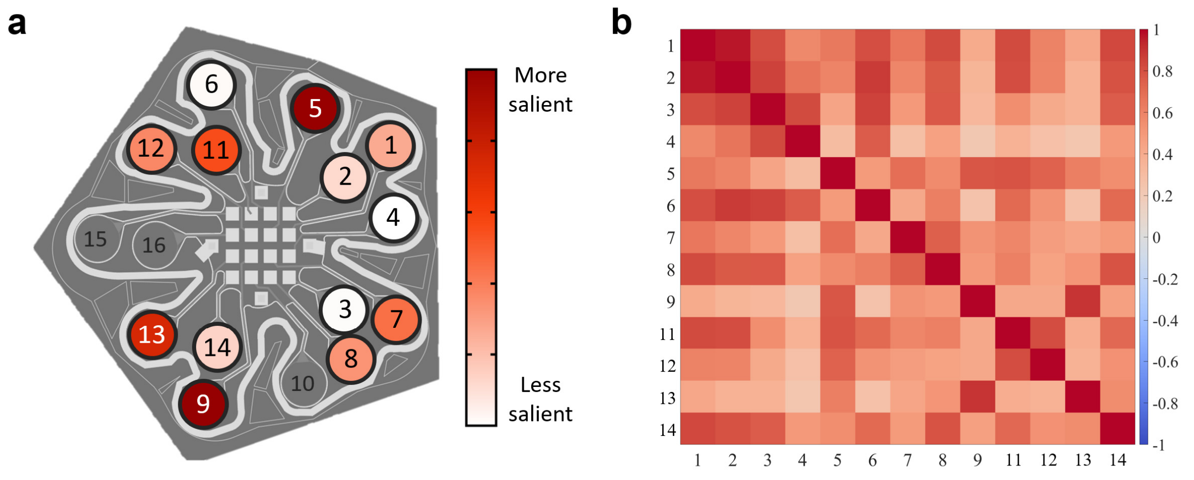

Supplementary Fig. 23

Neural activity discrimination across multiple IVI sites. a Significance distribution of discrimination accuracies across channels on the IVI, obtained from the normalized difference in the accuracy from each channel's data set relative to the accuracy from all data sets. **b** Pearson correlation coefficients matrix among IVI channels for the whole raw dataset (including three neural activity states).

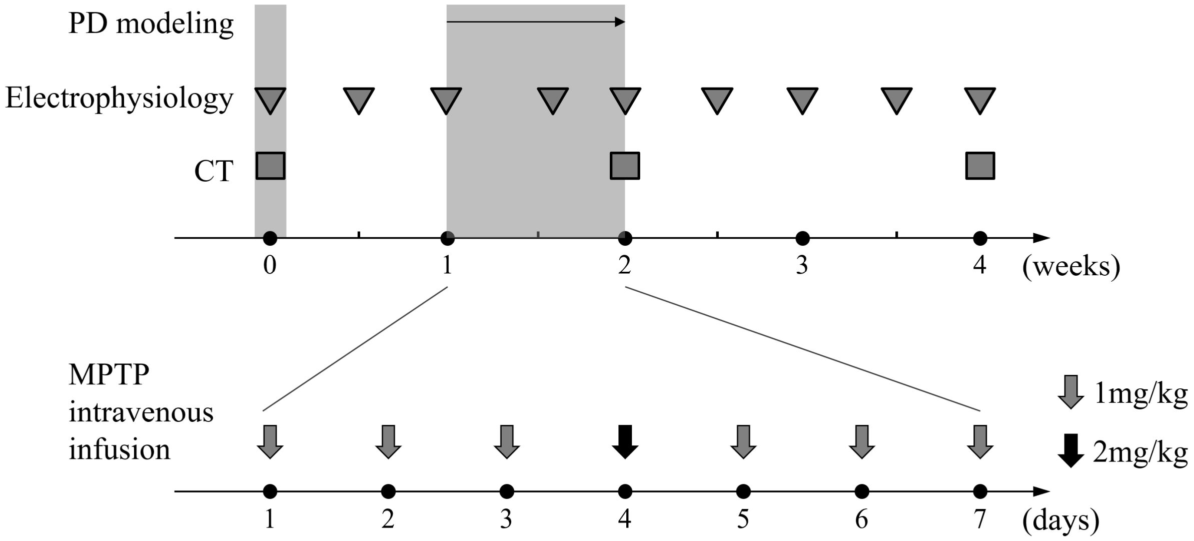

Supplementary Fig. 24
Experiment scheme of the in-vivo chronic recordings in parkinsonian sheep model.

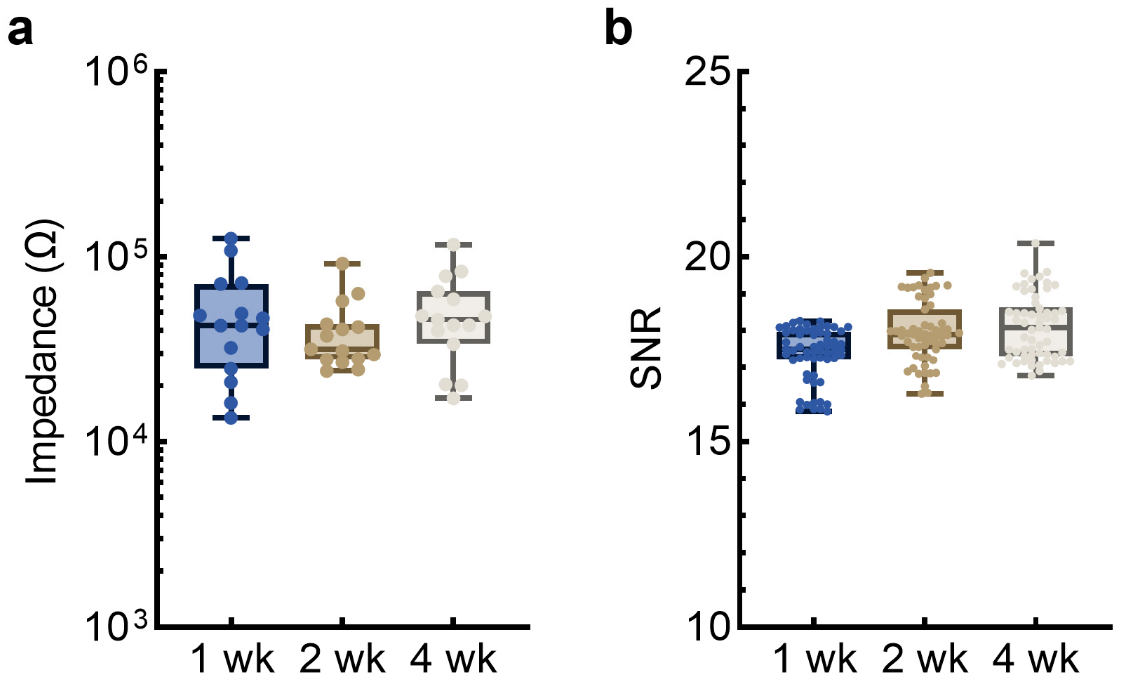

Supplementary Fig. 25

The fluctuation of the IVI channels' impedance and signal to noise ratio during in-vivo chronic experiment period. a Box plot showing the impedance fluctuation of the interface implanted in sheep for four weeks. n = 15 electrodes sites. **b** Box plot showing the signal-to-noise ratio fluctuation during the four-week recordings. n = 58 sessions of recordings. The box plots show the median and interquartile range, and the whiskers denote 1.5× the interquartile range. Source data are provided as a Source Data file.

References

1. Hu, J., Zhu, Y., Huang, H. & Lu, J. Recent advances in shape–memory polymers: Structure, mechanism, functionality, modeling and applications. *Prog. Polym. Sci.* **37**, 1720–1763 (2012).
2. Paulk, A. C. *et al.* Large-scale neural recordings with single neuron resolution using Neuropixels probes in human cortex. *Nat. Neurosci.* **25**, 252–263 (2022).
3. Murray, S. J. & Mitchell, N. L. The Translational Benefits of Sheep as Large Animal Models of Human Neurological Disorders. *Front. Vet. Sci.* **9**, (2022).
4. Darmani, G. *et al.* Long-Term Recording of Subthalamic Aperiodic Activities and Beta Bursts in Parkinson's Disease. *Mov. Disord.* **38**, 232–243 (2023).
5. Hammock, B. D. *et al.* A sheep model for MPTP induced parkinson-like symptoms. *Life Sci.* **45**, 1601–1608 (1989).
6. McCarthy, M. M. *et al.* Striatal origin of the pathologic beta oscillations in Parkinson's disease. *Proc. Natl. Acad. Sci.* **108**, 11620–11625 (2011).
