# Supplementary material for: Silk-enabled conformal intraventricular interfaces for minimally invasive neural recordings
Source: Nat Commun. 2025 Oct 23;16:9366. doi: 10.1038/s41467-025-64397-9 (PMC12549829; doi:10.1038/s41467-025-64397-9)
Supplement: Supplementary file 2 — Description of Additional Supplementary Files [file 41467_2025_64397_MOESM2_ESM.pdf]

Description of Additional Supplementary Files

Supplementary Movie 1: In vitro validation of minimally invasive implantation and conformal attachment of the IVI in the transparent lateral ventricle model.

Supplementary Movie 2: In vitro validation of controllable self-unfolding rate of IVI.

Supplementary Movie 3: In vitro validation of self-unfoldment on the concave model of IVI.

Supplementary Movie 4: In vitro validation of self-unfoldment on the convex model of IVI.
