# Supplementary material for: Silk-enabled conformal intraventricular interfaces for minimally invasive neural recordings
Source: Nat Commun. 2025 Oct 23;16:9366. doi: 10.1038/s41467-025-64397-9 (PMC12549829; doi:10.1038/s41467-025-64397-9)
Supplement: Supplementary file 7 — Reporting Summary [file 41467_2025_64397_MOESM7_ESM.pdf]

nature portfolio

Corresponding author(s): Zhitao Zhou

Last updated by author(s): Sep 13, 2025

Reporting Summary

Nature Portfolio wishes to improve the reproducibility of the work that we publish. This form provides structure for consistency and transparency in reporting. For further information on Nature Portfolio policies, see our Editorial Policies and the Editorial Policy Checklist.

Statistics

For all statistical analyses, confirm that the following items are present in the figure legend, table legend, main text, or Methods section.

n/a	Confirmed	
☐	☒	The exact sample size (*n*) for each experimental group/condition, given as a discrete number and unit of measurement
☐	☒	A statement on whether measurements were taken from distinct samples or whether the same sample was measured repeatedly
☐	☒	The statistical test(s) used AND whether they are one- or two-sided<br>*Only common tests should be described solely by name; describe more complex techniques in the Methods section.*
☐	☒	A description of all covariates tested
☐	☒	A description of any assumptions or corrections, such as tests of normality and adjustment for multiple comparisons
☐	☒	A full description of the statistical parameters including central tendency (e.g. means) or other basic estimates (e.g. regression coefficient) AND variation (e.g. standard deviation) or associated estimates of uncertainty (e.g. confidence intervals)
☐	☒	For null hypothesis testing, the test statistic (e.g. *F*, *t*, *r*) with confidence intervals, effect sizes, degrees of freedom and *P* value noted<br>*Give P values as exact values whenever suitable.*
☒	☐	For Bayesian analysis, information on the choice of priors and Markov chain Monte Carlo settings
☒	☐	For hierarchical and complex designs, identification of the appropriate level for tests and full reporting of outcomes
☐	☒	Estimates of effect sizes (e.g. Cohen's *d*, Pearson's *r*), indicating how they were calculated

Our web collection on statistics for biologists contains articles on many of the points above.

Software and code

Policy information about availability of computer code

Data collection	Electrophysiological signals were collected by a multichannel data acquisition system CereCube NSP8 (Neuroxess Co., Ltd., China). The 2D wide angle X-ray diffraction (2D-WAXD) was performed on a Xeuss 3.0 instrument (Xenocs, France). No custom software or code was used for data collection.
Data analysis	MATLAB2022b for electrophysiology data analysis and plotting data. MATLAB EEGLAB and Fieldtrip toolboxes were used. Prism (GraphPad Software, Version 9) for data analysis and plotting data. ABAQUS 2020 for mechanical simulations. COMSOL Multiphysics 6.0 software for electrostatic field simulation. The custom MATLAB scripts used for electrophysiology analysis are available from Zenodo (https://doi.org/10.5281/zenodo.16936239).

For manuscripts utilizing custom algorithms or software that are central to the research but not yet described in published literature, software must be made available to editors and reviewers. We strongly encourage code deposition in a community repository (e.g. GitHub). See the Nature Portfolio guidelines for submitting code & software for further information.

Data

Policy information about availability of data

All manuscripts must include a data availability statement. This statement should provide the following information, where applicable:

- Accession codes, unique identifiers, or web links for publicly available datasets
- A description of any restrictions on data availability
- For clinical datasets or third party data, please ensure that the statement adheres to our policy

The source data generated in this study are provided in the Source Data.xls file. The raw data for electrophysiology used for the analysis in this study are available from Zenodo (https://doi.org/10.5281/zenodo.16936239). Source data are provided with this paper.

Research involving human participants, their data, or biological material

Policy information about studies with human participants or human data. See also policy information about sex, gender (identity/presentation), and sexual orientation and race, ethnicity and racism.

Reporting on sex and gender	This is not relevant to our study.
Reporting on race, ethnicity, or other socially relevant groupings	This is not relevant to our study.
Population characteristics	This is not relevant to our study.
Recruitment	This is not relevant to our study.
Ethics oversight	This is not relevant to our study.

Note that full information on the approval of the study protocol must also be provided in the manuscript.

Field-specific reporting

Please select the one below that is the best fit for your research. If you are not sure, read the appropriate sections before making your selection.

☒ Life sciences ☐ Behavioural & social sciences ☐ Ecological, evolutionary & environmental sciences

For a reference copy of the document with all sections, see nature.com/documents/nr-reporting-summary-flat.pdf

Life sciences study design

All studies must disclose on these points even when the disclosure is negative.

Sample size	Sample size was estimated based on previous similar studies. [Perentos, N., Krstulovic, M. & Morton, A. J. Deep brain electrophysiology in freely moving sheep. Current Biology 32, 763-774.e4 (2022).] Chronic Parkinson's disease modeling was conducted in five sheep, acute intraventricular neural interface implantation in three sheep, long-term implantation in two sheep, and immunohistochemical analysis of the substantia nigra pars compacta in three sheep. Mouse and dog experiments were performed to validate the effectiveness of the planar shielding layer, with conclusions supported by within-animal comparisons across multiple devices and recording channels.
Data exclusions	No data were excluded from the analysis.
Replication	The number of repetitions for each experiment has been indicated in the manuscript and the legends of figures. Attempts at replication were successful and the conclusion were drawn from the analysis of multiple experiments.
Randomization	The animals were randomly chosen for disease modeling and implantation surgery.
Blinding	The investigators were not blinded to allocation during the intraoperative experiment and outcome assessment as the analysis of electrophysiological signals of parkinsonian sheep could not be performed without knowledge of the intraoperative condition of the sheep. We ensure that the data used to train models remained separate from the test data and the dataset splits were randomized.<br>The investigators were blinded to allocation during the chronic in vivo experiment with signals acquired by specialized experimental personnel.

Reporting for specific materials, systems and methods

We require information from authors about some types of materials, experimental systems and methods used in many studies. Here, indicate whether each material, system or method listed is relevant to your study. If you are not sure if a list item applies to your research, read the appropriate section before selecting a response.

Materials & experimental systems

n/a	Involved in the study	
☐	☒	Antibodies
☒	☐	Eukaryotic cell lines
☒	☐	Palaeontology and archaeology
☐	☒	Animals and other organisms
☒	☐	Clinical data
☒	☐	Dual use research of concern
☒	☐	Plants

Methods

n/a	Involved in the study	
☒	☐	ChIP-seq
☒	☐	Flow cytometry
☒	☐	MRI-based neuroimaging

Antibodies

Antibodies used	recombinant anti-tyrosine hydroxylase antibody (rabbit mAb) (targeting tyrosine hydroxylase, 1:500, Servicebio #GB15182-50, China), chicken anti-glial fibrillary acidic protein (GFAP) (targeting astrocytes, 1:1000, Abcam #ab4674, USA), goat anti-ionized calcium binding adaptor molecule 1 (Iba 1) (targeting microglia, 1:500, Abcam #ab5076, USA), and rabbit anti-neuronal nuclear (NeuN) (targeting nuclei of neurons, 1:1000, Abcam #ab177487, USA).
Validation	recombinant anti-tyrosine hydroxylase antibody (rabbit mAb) (Servicebio #GB15182-50) reacts with mammals.<br>Validation statement on the manufacturer's website and the analyzed immunohistochemistry provided in the manuscript:<br>https://www.servicebio.cn/goodsdetail?id=20765&specificationId=8251&specificationGroupId<br>https://www.novusbio.com/products/tyrosine-hydroxylase-antibody_nb300-109<br>The validation statement for the other three antibodies is based on previous research: Wei, S. et al. Shape-changing electrode array for minimally invasive large-scale intracranial brain activity mapping. Nat. Commun. 15, 715 (2024).

Animals and other research organisms

Policy information about studies involving animals; ARRIVE guidelines recommended for reporting animal research, and Sex and Gender in Research

Laboratory animals	Adult male C57BL/6 mice (10 weeks old) were used and were sourced from the Shanghai Laboratory Animal Research Center.<br>The adult male dog (Labrador, 3 years old) was used and was provided by Harborside Medical Technology Company (Shanghai, China), a laboratory animal institution accredited by China National Accreditation Service for Conformity Assessment (CNAS LA0026).<br>Five adult female sheep (Ovis aries, HuYang sheep, 1-2 years old, weighing 55-75kg) used in this study were obtained from and housed at the Harborside Medical Technology Company (Shanghai, China).
Wild animals	The study did not involve wild animals.
Reporting on sex	The sex of the sheep was considered based on previous similar studies. [Perentos, N., Krstulovic, M. & Morton, A. J. Deep brain electrophysiology in freely moving sheep. Current Biology 32, 763-774.e4 (2022).] For acute and chronic sheep intraventricular neural interface experiments, ewes were consistently used for electrode implantation due to practical surgical considerations, which is a common approach in sheep electrophysiology.<br>The sex of the mice and Labrador dog was not considered and was not associated with the findings and main conclusions of this study.
Field-collected samples	The study did not involve samples collected from field.
Ethics oversight	Ethical approval for our animal experiments involved mice was obtained from the Ethics Committee for Animal Management at the Shanghai Laboratory Animal Research Center, with approval number PA202300702.<br>The experimental protocol (IACUC-2023-019) for Labrador dog was approved by the Institutional Animal Care and Use Committee (IACUC) of Harborside Medical Technology Company (Shanghai, China), a laboratory animal institution accredited by China National Accreditation Service for Conformity Assessment (CNAS LA0026).<br>All surgical and experimental protocols for sheep were also approved by the IACUC of the Harborside Medical Technology Company (IACUC protocol 2023-090 and 2025-029) and were in accordance with The Association for Assessment and Accreditation of Laboratory Animal Care (AAALAC) International.

Note that full information on the approval of the study protocol must also be provided in the manuscript.

Plants

Seed stocks	This is not relevant to our study.
Novel plant genotypes	This is not relevant to our study.
Authentication	This is not relevant to our study.
